# Resequencing of the TMF-1 (TATA Element Modulatory Factor) regulated protein (TRNP1) gene in domestic and wild canids

**DOI:** 10.1186/s40575-023-00133-0

**Published:** 2023-11-15

**Authors:** James C. Sacco, Emma Starr, Alyssa Weaver, Rachel Dietz, Muhammad A. Spocter

**Affiliations:** 1 Ellis Pharmacogenomics Laboratory, College of Pharmacy and Health Sciences, Drake University, 50311 Des Moines, IA USA; 2 Department of Anatomy, Des Moines University, 50266 Des Moines, IA USA

**Keywords:** TRNP1, Cortical folding, Genetic polymorphism, Dog, Canidae

## Abstract

**Background:**

Cortical folding is related to the functional organization of the brain. The TMF-1 regulated protein (TRNP1) regulates the expansion and folding of the mammalian cerebral cortex, a process that may have been accelerated by the domestication of dogs. The objectives of this study were to sequence the *TRNP1* gene in dogs and related canid species, provide evidence of its expression in dog brain and compare the genetic variation within dogs and across the *Canidae*. The gene was located *in silico* to dog chromosome 2. The sequence was experimentally confirmed by amplifying and sequencing the *TRNP1* exonic and promoter regions in 72 canids (36 purebred dogs, 20 Gy wolves and wolf-dog hybrids, 10 coyotes, 5 red foxes and 1 Gy fox).

**Results:**

A partial TRNP1 transcript was isolated from several regions in the dog brain. Thirty genetic polymorphisms were found in the *Canis *sp. with 17 common to both dogs and wolves, and only one unique to dogs. Seven polymorphisms were observed only in coyotes. An additional 9 variants were seen in red foxes. Dogs were the least genetically diverse. Several polymorphisms in the promoter and 3'untranslated region were predicted to alter *TRNP1* function by interfering with the binding of transcriptional repressors and miRNAs expressed in neural precursors. A c.259_264 deletion variant that encodes a polyalanine expansion was polymorphic in all species studied except for dogs. A stretch of 15 nucleotides that is found in other mammalian sequences (corresponding to 5 amino acids located between Pro58 and Ala59 in the putative dog protein) was absent from the *TRNP1* sequences of all 5 canid species sequenced. Both of these aforementioned coding sequence variations were predicted to affect the formation of alpha helices in the disordered region of the TRNP1 protein.

**Conclusions:**

Potentially functionally important polymorphisms in the *TRNP1* gene are found within and across various *Canis* species as well as the red fox, and unique differences in protein structure have evolved and been conserved in the *Canidae* compared to all other mammalian species.

**Supplementary Information:**

The online version contains supplementary material available at 10.1186/s40575-023-00133-0.

## Abstract (plain language summary)

The folded shape of mammalian brains allows the cerebral cortex and other structures to attain a large surface area to fit into the skull. Over the past several thousand years, the domestication of dogs has led to increased folding as well as a great variety of skull size and shape amongst various breeds. Cortical folding, also known as gyrification, is regulated by numerous genes, including one that encodes the TMF-1 regulated protein (TRNP1). The TRNP1 protein in the brain affects the development of specialized cells in the brain that are involved in gyrification. It is not known whether a functionally distinct TRNP1 protein in dogs is responsible for the increased folding. Variations in the DNA sequence of the gene that encodes TRNP1 may be responsible for these dramatic changes in brain structure in dogs. This study sought to discover the differences in the TRNP1 DNA sequence in seventy-two canids, represented by thirty-six dogs of various breeds, twenty gray wolves and wolf-dog hybrids, ten coyotes, five red foxes and one gray fox.

After finding evidence of the expression of this gene in dog brain, we located thirty genetic changes or variants in the canids, with seventeen common to both dogs and wolves, and only one unique to dogs. Another seven of these genetic variants were observed only in coyotes. An additional nine variants were seen in red foxes. Dogs were the least genetically diverse species, an expected result of the inbreeding that characterizes domestication. Several of these changes may affect the function of the TRNP1 gene by affecting the binding of other biomolecules to regions in the DNA which regulate this gene. This study also found two other changes, one only found in dogs, and the other one only found in canids (compared to all other mammalian TRNP1 proteins) may change the length and three-dimensional structure and hence the function of the TRNP1 protein. This study concluded that numerous, potentially functionally significant dissimilarities in the TRNP1 gene exist between dogs and their wild relatives, as well as between canid and all other mammalian species.

**Supplementary Information:**

The online version contains supplementary material available at 10.1186/s40575-023-00133-0.

## Background

 The cerebral cortex is a crucial region in the mammalian brain that regulates cognitive behavior [22]. Cortical folding (also known as gyrification), which originates during fetal and early postnatal development, is intrinsically related to the functional organization of the brain and has long been studied as a marker of both normal and pathological brain function. In humans, cortical size is crucial for normal brain function, as patients with microcephaly or macrocephaly (in other words, small or enlarged brains, respectively) show a range of cognitive deficits. As part of the evolutionary process, cortical folding has enabled the mammalian brain to grow in volume and to expand in surface area despite being restricted to a certain skull size [58].

Based on cortical folding, mammals can be divided into lissencephalic species (such as mice and manatees), which have smooth-surfaced cortices, and gyrencephalic species (such as ferrets and small primates), which exhibit convolutions in the cortex [23]. Typically, gyrencephalic brains are found in large rodents and large primates. The gyrification index (GI) is a measure of the total cortical surface area relative to the convex smooth hull that defines the outer boundaries of the cerebrum. Across mammalian species, including carnivores and canids, the GI shows a strong positive relation with brain mass [18, 37, 43].

Artificial selection has resulted in domestic dog (*Canis lupus familiaris*) breeds that have diverged significantly from the form of their closest ancestor, the grey wolf (*Canis lupus lupus*). While brain size has decreased by about 30%, the GI in dogs has not decreased [18, 56]. A brain imaging study conducted on wild canid and domestic brains concluded that the GI in canids is positively correlated with cortical surface area, thickness and total gray matter volumes [18]. However, within domestic dogs the strength of this hypoallometric relationship is dramatically reduced. In addition, the hyperallometric relationship displayed between cortical thickness and GI for the *Canidae* indicates that global folding changes outpace changes in the underlying cortical thickness. In addition, lower local GI (i.e., regional gyrification measured within a defined functional area) in foxes compared to dogs, wolves and coyotes imply a divergent folding pattern for the anterior temporoparietal area between “fox-like” and “wolf-like” canids, [18]. The domestic dog also exhibits more morphological diversity than any other species, with the greatest variation evident in the size and shape of the skull [55], which in turn has led to distinct changes in cerebral organization between dolichocephalic (long-skulled) and brachycephalic (short-skulled) breeds [44].

The mammalian TMF-1 (TATA Element Modulatory Factor) regulated protein (*TRNP1*) is one of several genes that are known to regulate the expansion and folding of the mammalian cerebral cortex by accelerating cell cycle progression [54]. This gene is expressed in the ventricular zone and neuronal layers of the developing cortex. TRNP1 levels have contrasting effects on the expansion of the murine cerebral cortex, with high expression in the germinal layers of the precentral and parahippocampal gyri promoting neural stem cell self-renewal and tangential expansion, whereas low expression in the germinal layers of the occipital and temporal lobes induces radial expansion and increased gyrification [52]. Recently, it has been demonstrated that TRNP1 acts as a negative transcriptional regulator of basal progenitor genesis, controlling radial glial fate by its interaction with several nuclear membrane-less organelles [11].

It is not known whether the changes observed in the dog brain due to domestication are a result of altered expression of *TRNP1* and/or sequence differences within the TRNP1 protein that alter its function. As with other proteins, the level of TRNP1 in the brain is subject to interindividual variation, which can be due to various extrinsic or intrinsic factors. An important intrinsic factor is the presence of genetic polymorphisms in the *TRNP1* gene, which may account for differences in gyrification observed within dogs and across the *Canidae*.

Therefore, we designed a study whose objectives were to sequence the gene coding for TRNP1 in both a diverse dog population and selected wild canid species, screen for genetic polymorphisms, and predict any effects these variants may have on TRNP1 function. We also aimed to provide evidence of its expression in select regions of the canid brain. Finally, we also compared the experimentally determined TRNP1 DNA and resulting inferred protein sequences in domestic dogs with published TRNP1 sequences for other canids and carnivores as well as species representative of other mammalian orders.

## Methods

Animals and sample collection. A total of 72 canids were included in this study. Thirty-six pure-bred dogs (representing 33 *Canis lupus familiaris* breeds, including 2 dingoes and 3 New Guinea singing dogs) recruited for this study were either privately owned or provided through the collaboration of the Jordan Creek Animal Clinic, West Des Moines IA (Additional file 1). The American grey wolves (*Canis lupus lupus*, *n* = 20), coyotes (*Canis latrans*, *n* = 10), and foxes (red fox *Vulpes vulpes*, *n* = 5, gray fox *Urocyon cinereoargenteus*, *n* = 1) were from the Colorado Wolf and Wildlife Center (Divide, CO), Wolf Park (Battle Ground, IN), Shy Wolf Sanctuary (Naples, FL), Blanke Park Zoo (Des Moines, IA), and the JAB Canid Education and Conservation Center (Santa Ysabel, CA). Buccal cell samples were collected from the dogs using cheek swabs designed for use in canines (Performagene®, DNA Genotek Inc.). DNA collected in this manner is stable for at least one year when stored at room temperature. Brain tissue was obtained from a single dog that was euthanized due to terminal illness. All experimental procedures were approved by the Drake University Institutional Animal Care and Use Committee.

Identification of target region. Since the chromosomal location of *TRNP1* is unannotated in the most recent version of the canine genome (canFam6), we located the predicted transcript (XM_038462447) using the gene search function on the UCSC Genome Browser. To ensure that this transcript represents TRNP1 mRNA and also to identify exons, we then used the blastn tool (available at: https://blast.ncbi.nlm.nih.gov/Blast.cgi) to search for highly similar sequences (megablast) in the mammalian nucleotide and *Canis lupus familiaris* EST collection while excluding predicted transcripts. Having established the identity of the predicted transcript as TRNP1, we used the BLAT Search Genome Tool [25] to align the sequence with the canine genome assembly. We also used the Broad Improved Canine Annotation v1 track data hub within the UCSC Genome Browser [20]) to provide evidence of tissue expression of the TRNP1 transcript.

### Nucleic acid isolation, gDNA and cDNA amplification and gene resequencing

Following inactivation of nucleases and precipitations of impurities in the buccal samples, genomic DNA (gDNA) was purified via ethanol precipitation (Performagene®, DNA Genotek Inc.). We used 200 ng of genomic DNA to amplify both exons and the proximal promoter region of TRNP1. The reaction mixture consisted of a 25 µl of reaction mixture containing 0.25 µM of each primer (Table 1), 4–8% of GC Enhancer, and Amplitaq Gold 360 Master Mix (Applied Biosystems, Foster City, CA). After an initial incubation at 95 °C for 10 min, PCR amplification was performed for 40 cycles consisting of 95 °C for 30 s, 60 °C for 30 s and 72 °C for 45 s, followed by a final extension at 72 °C for 7 min. The specificity of each PCR was checked by electrophoresis on a 1.5% agarose gel.

**Table 1:** Sequences of primers used for the amplification of the *TRNP1* gene and transcript

Primer name	Oligonucleotide sequence (5`→3`)	Region/length amplified
Primers for gDNA template		
cfTRNP1_prom_F cfTRNP1_prom_R	TGGGTCTGTTTCCTCCTCTG CGGAGTCAAGGTCGGAGTT	Proximal promoter/exon 1 c.-829 to c.135
cfTRNP1_ex1_1F cfTRNP1_ex1_2R	CGTCGGTCTTCCCCCTCG CGCTGGCCCTTCTTGAGG	Exon 1/Coding sequence c.-76 to c.*94
cfTRNP1_ex2_F cfTRNP1_ex2_R	AAGAGTTTGGGTGGGGTTGG TGCGGAATACAAGTTCTGGGT	Exon 2/3`UTR c.*143 − 169 to c.*802
Primers for cDNA template		
cfTRNP1_839F cfTRNP1_968R	CGGGAAGAGACAGTCGGG TGTTTAAGGTTGTGGGACGTC	Partial transcript (within 3`UTR) c.*97 to c.*246
cfTRNP1_868F cfTRNP1_1445R	CAGTGAAGGAGGCTGCTC CTCCCTGAGAATGAGGTGCC	Partial transcript (within 3`UTR) c.*126 to c.*713

Positions are denoted with respect to the predicted start codon. 3’UTR: 3’untranslated region; underlined nucleotides indicate exon 1/2 splice junction; * indicates numbering from start of 3`UTR

Total RNA was isolated from distinct regions (cerebellum, hippocampus, occipital cortex, frontal cortex) within a single dog brain (Purelink RNA Mini Kit,) and 2 $\mu$g was reverse transcribed into first-strand cDNA using the High-Capacity cDNA Reverse Transcription Kit (Applied Biosystems). Two sets of primers were used in the subsequent PCR, each designed to amplify progressively longer stretches of the TRNP1 transcript (Table 1). The PCR conditions were similar to those described above, except for the exclusion of GC enhancer.

Following purification by Exo-SapIT (Affymetrix, Santa Clara, CA), the amplicons were submitted to bidirectional Sanger sequencing using the Big-Dye Terminator v3.1 (Eurofins Genomics, Louisville, KY). 5% of the samples were randomly chosen and resequenced to confirm the initial genotype result. Sequence assembly and identification of genetic polymorphisms was performed using Staden package software (http://staden.sourceforge.net/).

Data analysis. In order to determine the degree of conservation between the canid species, DNA sequences were compared using ClustalOmega [50]. The aligning of transcription factors to the canid *TRNP1* promoter sequence was performed by using JASPAR position-specific scoring matrix models through the LASAGNA algorithm v 2.0, an integrated webtool for transcription factor binding site search and visualization [29, 30]. By inputting sequence variants, we identified the effects that promoter polymorphisms may have on DNA binding proteins that may bind to our sequence of interest. Further information on the function of these transcription factors was obtained from UniProtKB [53] and their potential relevance to the role of TRNP1 in cortical development assessed using GO annotations available at the Gene Ontology Resource [4, 17], specifically “cerebellar cortex morphogenesis” (GO:0021696), “interkinetic nuclear migration” (GO:0022027) and “neural precursor cell proliferation” (GO:0061351). The effect of 3’UTR genetic polymorphisms on microRNA (miRNA) binding was analyzed by submitting sequence variants to the MirTarget target prediction tool available at the miRDB database [9, 32]. Sequence, expression and functional information on the miRNAs of interest was obtained from the miRmine database [42] and TargetScan prediction tool [39]. The MPI bioinformatic toolkit [15] and the Phyre2 protein fold recognition server [24] were used to subject the predicted dog TRNP1 protein (XP_038318375) to sequence analysis, prediction of secondary structure, and sequence comparison with its homologs from the genus Carnivora (8 species from the *Canidae* and 32 species from other families) and with 25 other species, representative of six other mammalian genera. Since no TRNP1 experimental protein structure is available, we used the HHPred tool to select homologous templates of known structure and forwarded the alignment to Modeller [45] in order to calculate a modeled structure for the dog TRNP1 protein.

The effect of genetic variation on the structure and function of the TRNP1 protein within and across species were predicted by the following tools: Polyphen-2 [1], Align GVDG [38] Coils [35] Deepcoil [34], and Phyre2 [24].

## Results

The 1,682 nucleotides of the predicted XM038462447.1 transcript align with 100% identity to dog chromosome 2, at locus 69,744,731 − 69,751,120. The gene is composed of two exons (ex 1: 907 bp; ex 2: 774 bp), separated by a single intron (4827 bp). Experimental evidence for the entire sequence was inferred from partial mRNAs (all incomplete at the 5’ end), recorded as canine expressed sequence tags (ESTs) isolated from kidney, ovary, heart, muscle, and cerebral cortex (https://genome.ucsc.edu). Only the sequence of one EST (CF411103) includes the single splice junction. The predicted full-length transcript aligned with several primate and rodent TRNP1 reference sequences. The closest match (79.23% identity) was with pig TRNP1, represented by NM_001243828. Very poor identity was obtained with non-mammalian vertebrate sequences, as reported by other studies [54].

Despite multiple attempts at optimization, we were unable to isolate the entire TRNP1 transcript (predicted length 1682 bp), probably due to the high GC content in exon 1 (80%). However, we were able to sequence a partial length (606 bp) TRNP1 transcript from all brain tissue samples, that is, cerebral cortex, hippocampus, frontal cortex and, cerebellum. This sequence (represented by GenBank accession numbers OQ191934, OQ191935) corresponds to 78% of the 3` UTR region that spans both exons. The regions of *TRNP1* that were sequenced using gDNA as template exhibited 95% or higher similarity across the five species investigated, with a higher degree of conservation in both exons (97.8% or higher) than in the proximal promoter (95.67 or higher).

**Fig. 1:**
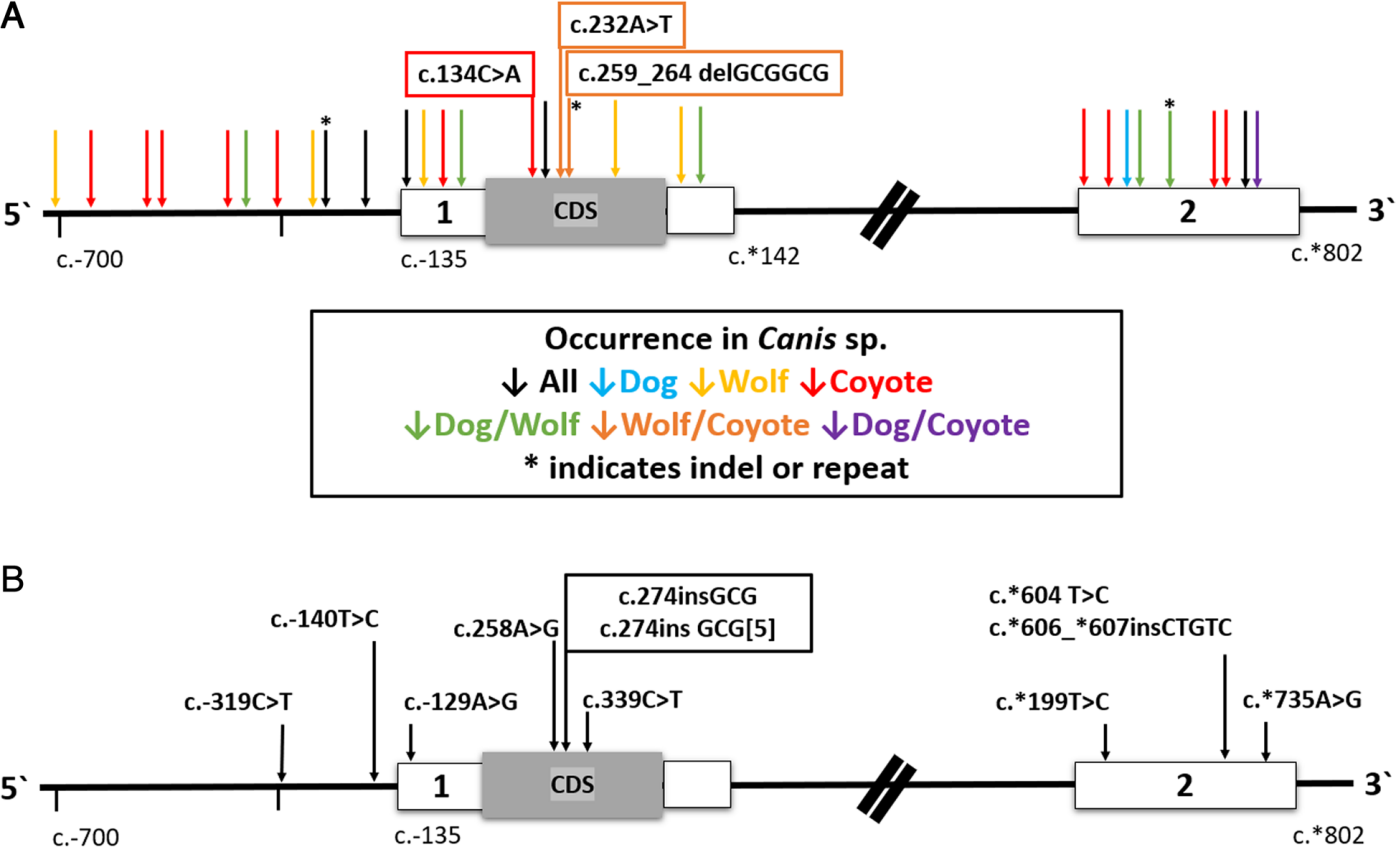
*TRNP1* genetic polymorphisms detected in this study. **A** *Canis* species; **B** Red fox. Boxed polymorphisms indicate nonsynonymous variants. Intron 1 not to scale

 Thirty TRNP1 polymorphisms were identified within the dog, wolf and coyote cohorts, with dogs and coyotes being the least and most genetically diverse respectively. All variants represented single base substitutions, with the exception of c.-240delA in the proximal promoter (rs852983207), a GCGGCG deletion in the coding region of exon 1 at c.259_264 (resulting in p.Ala87_Ala88del), and a variable TG dinucleotide repeat (*n* = 1–3) in exon 2 at c.*370_371 (Fig. 1A). Significant sequence similarity enabled us to use the same primers to amplify the homologous *TRNP1* regions in the fox species. This enabled us to identify 9 polymorphisms in the red fox, three of which (c.-129 A > G, c.274insGCG, c.274insGCG [5]) occur at analogous loci in the *Canis* species (Fig. 1B, Additional file 2).

**Fig. 2:**
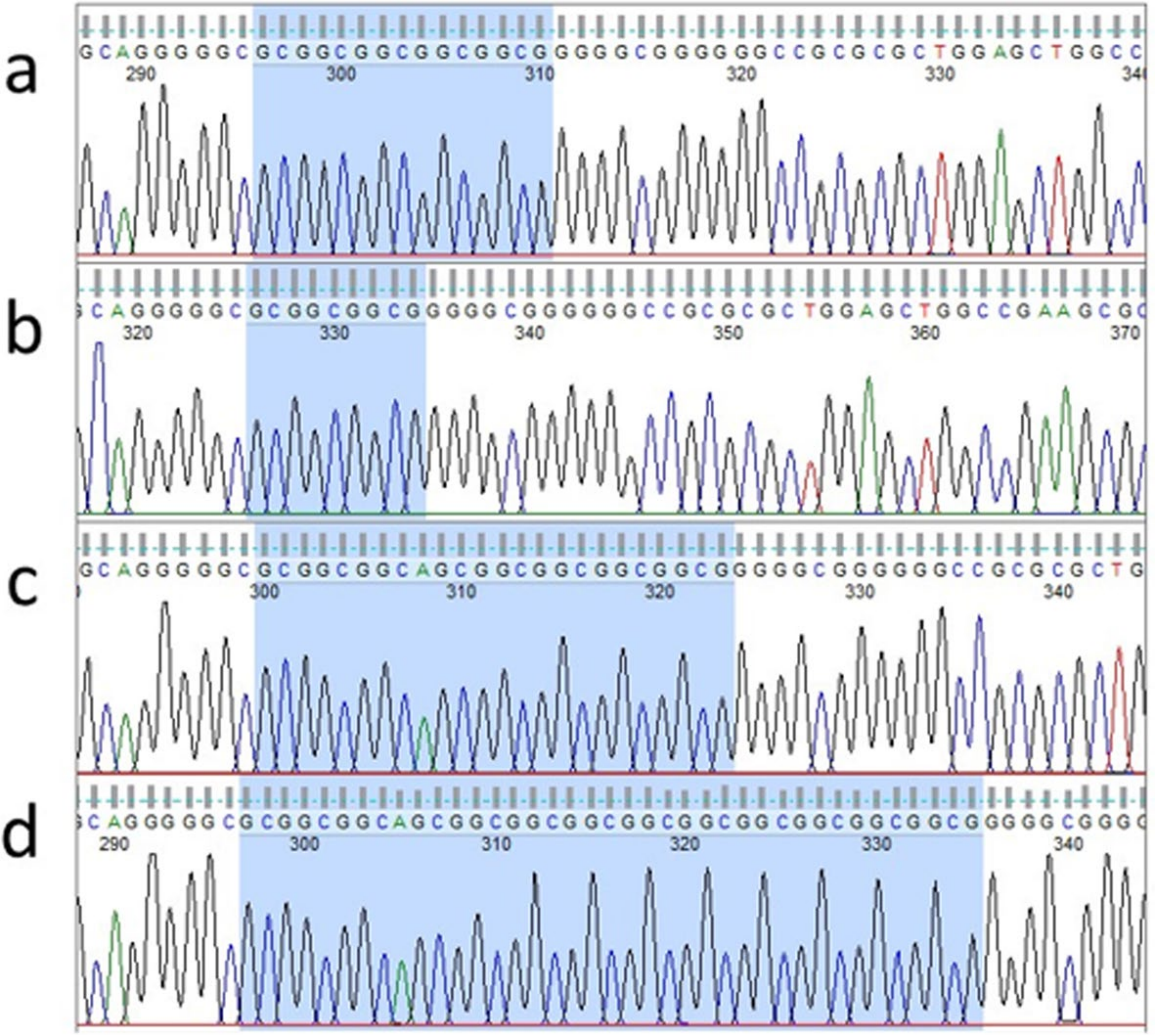
DNA electropherograms representing the alignment of the GCG repeat polymorphism in (**a**) dog, (**b**) wolf, (**c**, **d**) red fox

Comparison of the sequences of the deletion variant at c.259_264 across the five canid species indicates that this results in a trinucleotide GCG repeat polymorphism that occurs within wolf, coyote and red fox populations (Fig. 2). This locus was not polymorphic in dogs, who have a strongly conserved consecutive series of five GCG repeats, representing 5 alanine residues (AAAAA). At least 35% of coyotes and wolves possessed the allele represented by three GCC repeats (AAA). In red foxes, three alleles were found, represented by 8 ((GCG)_2_GCA(GCG)_5_), 9 ((GCG)_2_GCA(GCG)_6_) and 13 ((GCG)_3_GCA(GCG)_9_) repeats that encode variable polyalanine lengths. The single grey fox sequenced had a series of 9 consecutive GCG repeats.

**Fig. 3:**
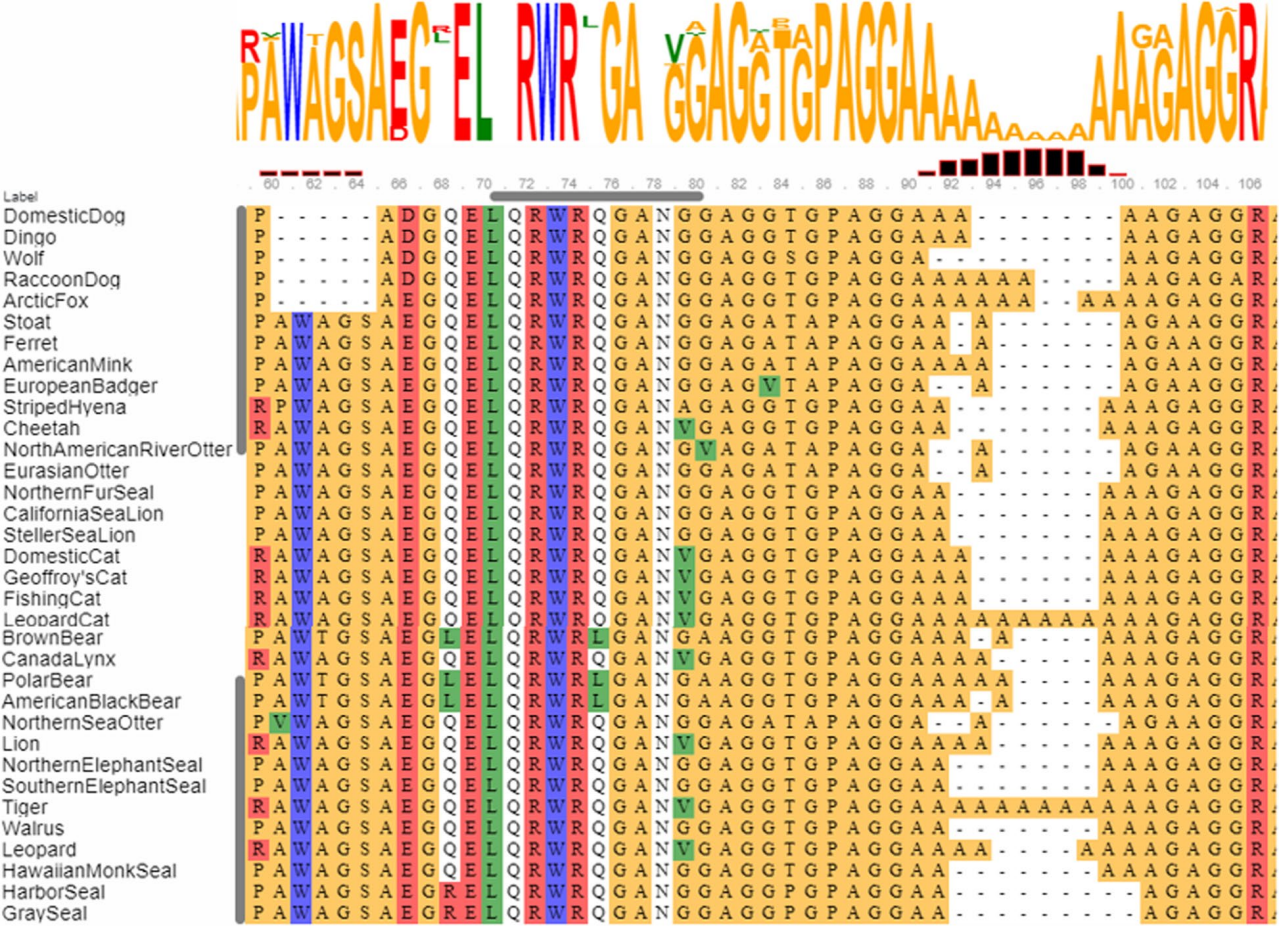
Clustal Omega alignment of part of the *N*-terminus of the TRNP1 protein in 34 carnivore species. The variable alanine repeat is shown to the left. Color coding for individual amino acids is as follows: yellow – small/polar; red – charged; blue - aromatic; green - hydrophobic

This polyalanine region appears to be very variable across the *Carnivora*, with the number of consecutive alanine repeats ranging from 3 in wolves to 12 in tigers and leopard cats, in an otherwise highly conserved part of the protein (Fig. 3). A similar degree of variability was observed in 43 additional species representing 9 additional mammalian orders (Additional file 3).

**Fig. 4:**
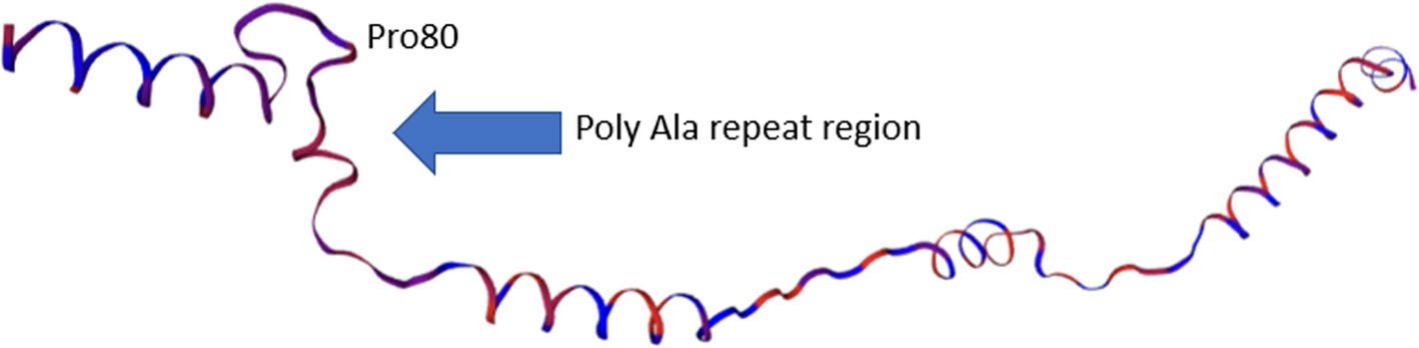
Calculated model for the *N*-terminal region of the dog TRNP1 protein (residues Ala59-Ala166 shown), based on homology with the cryo-EM structure of the MukBEF monomer (PDB#7NYY)

Homology modeling predicted that this polyalanine repeat lies between two alpha helices in the *N*-terminal disordered region (Fig. 4). A highly conserved proline at position 80 in the dog (position 87 in the consensus sequence in Fig. 3) creates a kink in the protein chain which separates two alpha helices. This accuracy of this prediction was limited by the fact that only 40% of the sequence could be modelled with high confidence due to the disordered region.

**Fig. 5:**
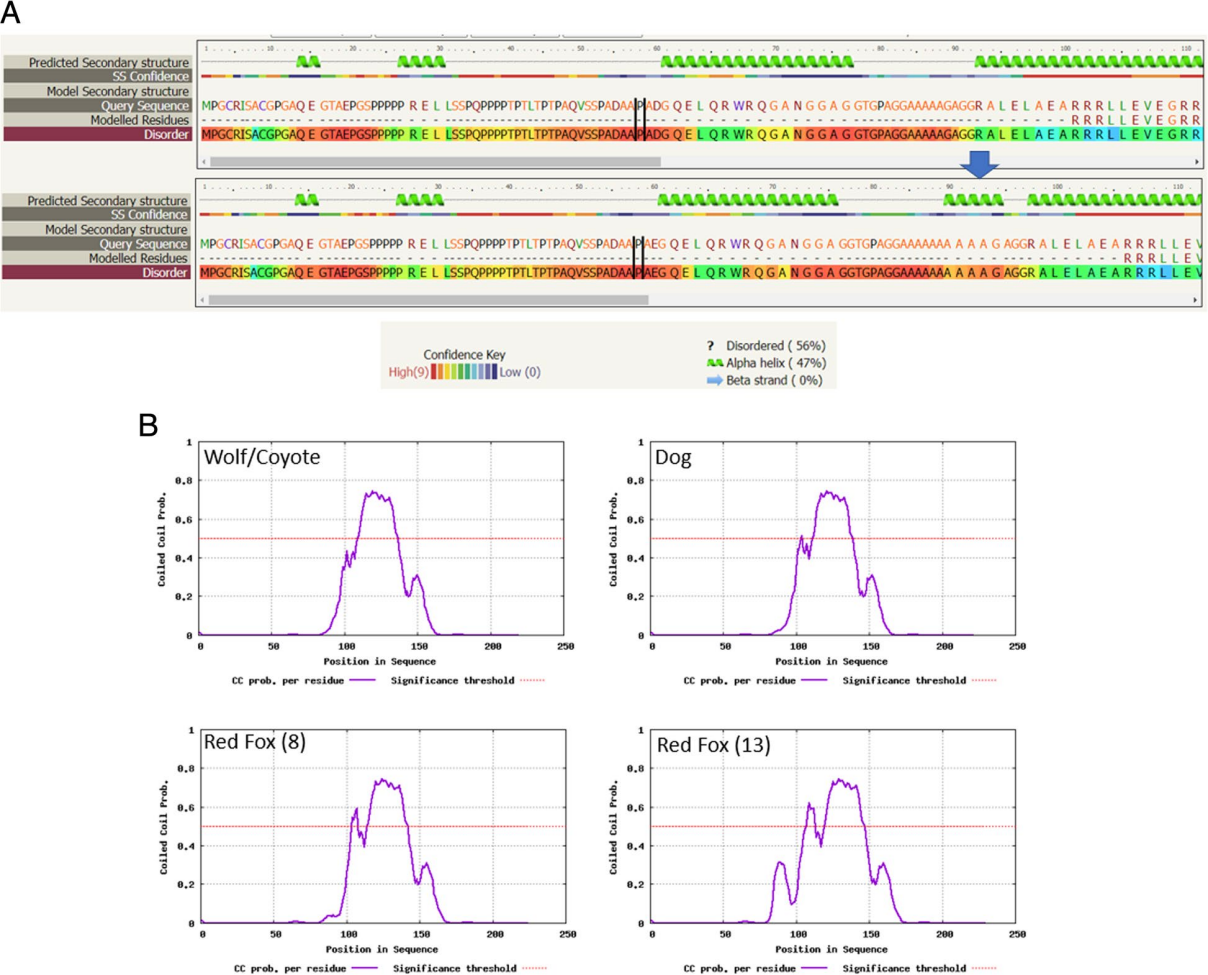
Comparison of the effect of polyalanine repeats on TRNP1 protein secondary structure between dog and wild canids. **A** Alpha helix formation (top: dog; bottom: red fox (Ala_10_)*) as predicted by Phyre2 with Pro80 highlighted by parallel lines; **B** Probability of formation of coiled coil structure in wolf/coyote (Ala_3_), dog (Ala_5_) and red fox (Ala_8,13_) as calculated by DeepCoil. *length representative of alleles observed in red fox

 Regardless of species, the formation of alpha helical structures is predicted to occur in this part of the protein if the number of consecutive polyalanine repeats exceeds 7, as for example, is the case with red fox (Fig. 5A). Due to the disordered nature of the region, this prediction is, however, of low confidence. Additionally, an increased number of polyalanine repeats may affect the structure and stability of the coiled coil domain, which is located just downstream of the repeat region (Fig. 5B).

There were distinct differences in the allele frequency of several polymorphisms between the wolves and dogs in the study (Fig. 1A; Table 2). Thirteen variants occurred in two or all three species, while others were only detected in one species. For example, while domestic dogs share 11 polymorphisms with one or all canid species, only one mutation was specific to dogs (c.*280 C > A). Conversely, five polymorphisms were unique to wolves and another 11 variants were only found in coyotes. The variants represented by c.-240delA, the c.-412 C > T, and the c.*370_371insTG appear to be the major allele in dogs with respect to the reference allele currently represented at the same locus in canFam6.

**Table 2:** Genetic polymorphisms in the canid *TRNP1* gene, variant allele frequencies (AF) and predicted effect of variants on regulatory and coding regions

Gene region	Genetic polymorphism	Variant AF D W C	Predicted effect	Known function or expression of affected transcription factor or miRNA in brain tissue
Promoter	c.-702 C > G	0.00 0.03 0.00	TCF3, NFIC binding site deleted	Both genes expressed in cerebral cortex. TCF3 is a transcriptional regulator involved in the initiation of neuronal differentiation and mesenchymal to epithelial transition
	c.-665 C > T	0.00 0.00 0.10	KLF4 binding site deleted; New NR2E3 binding site	KLF4 regulates the expression of key transcription factors during embryonic development. NR2E3 involved in cerebellar cortex morphogenesis
	c.-607 T > C	0.00 0.00 0.05	None	
	c.-584 G > A	0.00 0.00 0.10	New CTCF binding site	Negative regulation of cell population proliferation
	c.-444 C > A	0.00 0.00 0.05	MYC binding site deleted; New TBXT, ZEB1 binding sites; Increased binding for EN1	MYC expressed in the proliferating cells of the developing CNS. EN1 and TBXT involved in cerebellar cortex and neural plate morphogenesis. ZEB1 positively regulates neuronal differentiation.
	c.-412 C > T	0.60 0.15 0.00	New ZFX binding site	None reported
	c.-355 C > T	0.00 0.00 0.05	New PAX5, PAX6 binding sites; Increased binding for RUNX1	PAX important for cerebellar cortex morphogenesis, including regulation of neuroblast proliferation and neuron migration. RUNX1 involved in neuron differentiation.
	c.-256 G > C	0.00 0.03 0.00	ELK1, MIZF binding sites deleted	ELK1 Is a positive regulator of neuron death while MIZF is involved in cerebellar cortex morphogenesis.
	c.-240delA	0.76 0.38 0.05	REST, ZNF423 binding sites deleted	Expressed in neural progenitor cells, REST is a transcriptional repressor that regulates neurogenesis and neuron differentiation. ZNF423 regulates cerebellar granule cell precursor cell proliferation.
	c.-161 T > G	0.04 0.10 0.10	SpI1 binding site deleted; Increased binding for NFYA	None reported
5’UTR	c.-129 A > G	0.13 0.08 0.10	New binding sites for TBP	None reported
	c.-104 T > C	0.00 0.03 0.00	None predicted	
	c.-79 C > T	0.00 0.00 0.10	New binding sites for ARNT and CREB1	CREB1 involved in axonogenesis
	c.-58 C > T	0.25 0.15 0.00	None predicted	
Coding	c.134 C > A	0.00 0.00 0.05	p.(Pro45Gln) – probably damaging	
	c.141 T > G	0.39 1.00 0.85	p.Pro47=	
	c.232 A > T	0.00 0.43 0.25	p.(Thr78Ser) – probably benign	
	c.259_264 delGCGGCG	0.00 0.48 0.35	p.(Ala87_Ala88del) – may alter spacer length	
	c.453 G > A	0.00 0.03 0.00	Gln151Gln	
3’UTR	c.*19 A > G	0.00 0.05 0.00	None	
	c.*41 G > T	0.08 0.13 0.00	None	
	c.*149 C > T	0.00 0.00 0.05	None	
	c.*257 G > A	0.00 0.00 0.15	New sites for hsa-miR-335-3p, 450b-3p, 769-3p, 5694	hsa-miR-335, 769 expressed at high levels in human brain
	c.*280 C > A	0.03 0.00 0.00	None	
	c.*286 A > G	0.13 0.23 0.00	None	
	c.*370_371insTG	0.64 0.95 1.00	Deletion of hsa-miR-128, 216a, 3681 sites	cfa-miR-128-1 detected in canine cerebrospinal fluid; hsa-miR-128-3p, 216a-3p expressed at high levels in human brain
	c.*452 T > A	0.00 0.00 0.05	None	
	c.*454 A > G	0.00 0.00 0.05	None	
	c.*482 C > T	0.01 0.28 0.05	Deletion of hsa-miR-549a-3p site	
	c.*495 C > G	0.03 0.00 0.55	Deletion of hsa-miR-6820-3p, hsa-miR-3164 sites	

*D* dog; *W* wolf; *C* coyote. Alleles are denoted as variant with respect to the reference allele at that locus on the (+) strand on the canFam6 assembly. Variant accession numbers are given in Additional file 2. * indicates numbering from start of 3'UTR. Software prediction programs listed in text. Function of transcription factors were based on gene ontology using the GO: biological process annotation

Several polymorphisms in the TRNP1 proximal promoter were predicted to alter the binding of transcription factors by deleting or altering the interaction with existing sites as well as creating novel sites (Table 2). A number of these transcription factors play important roles in cerebellar cortex morphogenesis, including RE1-silencing transcription factor (REST), homeobox protein engrailed-1 (EN1), transcriptional repressor CTCF (C2CF) and zinc finger protein 423 (ZNF423). Four genetic polymorphisms in the 3’UTR region were predicted to delete or create new binding miRNA sites (Table 2). Several of the miRNAs that may be impacted, such as hsa-miR-335-3p, hsa-miR-769-3p, and hsa-miR-216a, are expressed at high levels in the human brain.

Only one polymorphism, the synonymous variant c.141 T > G, was found in the TRNP1 coding region in dogs; the variant allele G was the only or major allele in wolves and coyotes respectively. Another coding SNP, c.232 A > T (Thr > Ser78), found in wolves and coyotes, is predicted to be benign (Polyphen-2 score: 0.000; align GCVG Class C55). Conversely, another nonsynonymous polymorphism, c.134 C > A, which leads to the amino acid change Pro > Gln45, found in a single coyote, was predicted to be damaging (Polyphen-2 score: 0.988; align GCVG Class C65), as the proline at that locus is highly conserved in mammals.

**Fig. 6:**
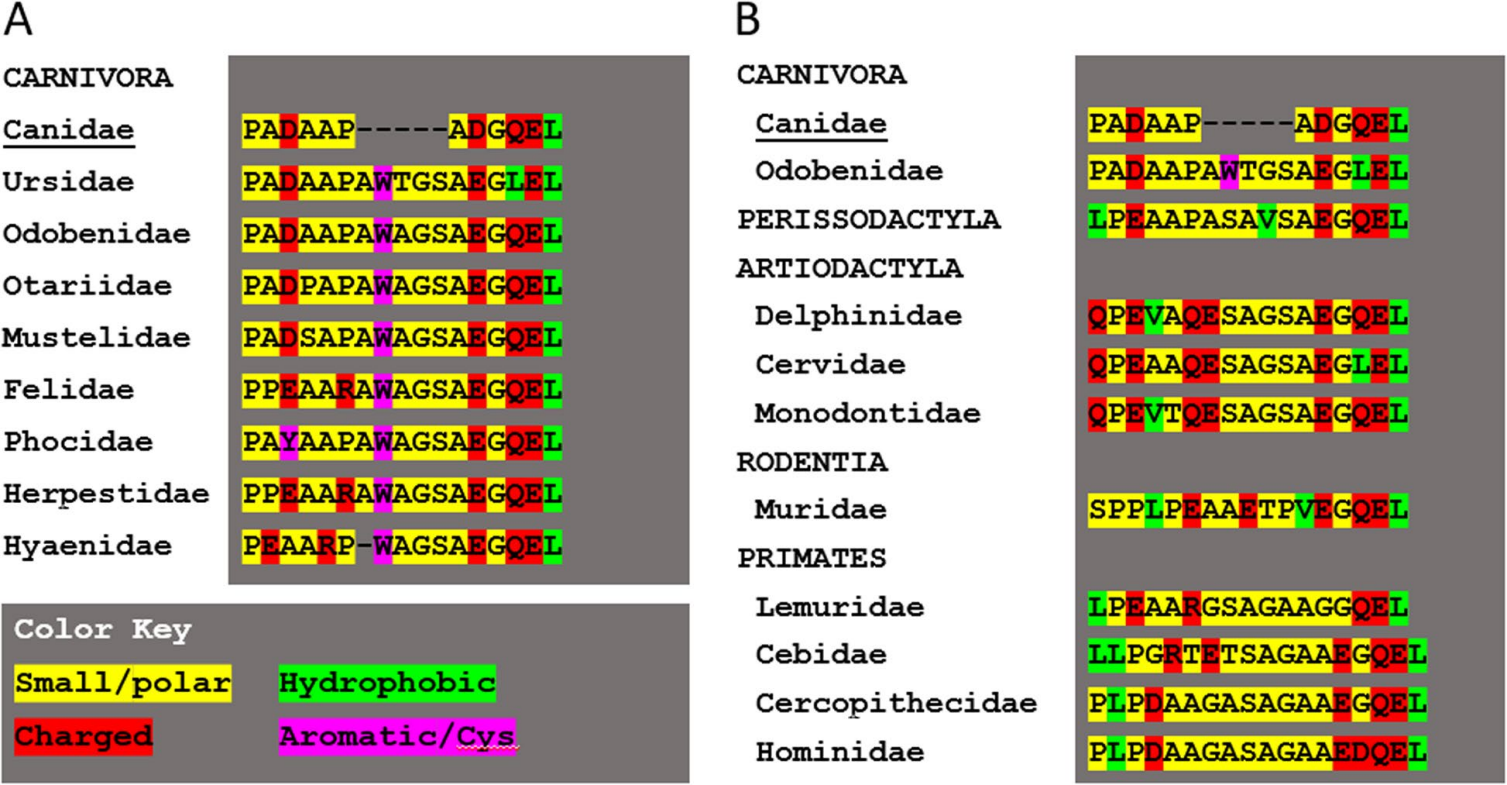
Comparison of the sequence that comprises the five amino acid gap in the N-terminal region of the TRNP1 protein that is unique to the *Canidae*. Alignment with: **A**. Other families included in the order Carnivora, **B**. Other mammalian orders. Residue size, charge and water solubility are color-coded. The protein sequences used to align with the dog sequence XP_038318375 were representative species listed in Additional file 3

All the canid TRNP1 DNA sequences determined by this study, as well as the predicted nucleotides for two others (common raccoon dog *Nyctereutes procyonoides*, and Arctic fox *Vulpes lagopus*), exhibit a distinctive 15-base pair gap in the coding region (represented by the absence of 5 amino acids located between Pro58 and Ala59 in dogs), when compared to all other mammalian TRNP1 sequences, including other species of the order *Carnivora* (Fig. 6). Species belonging to the *Canidae* lack an A/PWA/TGS sequence that appears to be ubiquitous to other carnivores. The end-result of this gap is a smaller stretch of small or polar amino acids, that is flanked, as with all other species by charged residues such as glutamine, aspartic and glutamic acids.

## Discussion

Experimental sequencing and comparative genomic tools enabled us to confirm that, as with other mammals, the canid *TRNP1* gene, located on dog chromosome 2, is composed of two exons. Exon 1 and the proximal promoter region are located within a 1 kb CpG island, indicating a housekeeping gene function for TRNP1. Although there is no evidence of methylation of individual CpG sites in dog brain or placental tissue [46, 47], the technique used so far is limited by its low coverage and a detailed study of the area is likely to reveal evidence of epigenetic alterations. The entire coding region (666 bp in dogs) lies within the first exon and constitutes only 40% of the transcript, while the larger 3’UTR region is comprised within both exons. Disproportionately large 3’UTRs are often observed in genes that encode proteins that are involved in multiple protein interactions, and the nervous system selectively expresses isoforms with longer 3’UTRs [5].

The high GC content described above was likely the reason for our failure, despite extensive PCR optimization, to amplify the full, or at least the 5’ end of the dog TRNP1 transcript. In fact, a number of mammalian genomes with low coverage also exhibit gaps in their homologous exon 1 regions. Nevertheless, we were able to isolate around 90% of the 3’UTR, including the region that comprises the splice junction. This transcript was present in all four dog brain regions investigated.

Amongst the *Canis* species, coyotes were observed to have the greatest number of genetic polymorphisms, 11 out of 20 (or 55%) being unique to that species. Similarly, 5 variants were observed exclusively in wolves, representing 45% of the total number of polymorphisms found in this wild canid. Conversely, dogs, even though they represented various breeds, including ancestral breeds such as dingoes and New Guinea singing dogs, had the lowest number of polymorphisms (6), with only one mutation being specific to dogs. This lower genetic diversity is a hallmark of domestication, which may also explain the wide differences in allele frequency observed for some variants (c.-412T, c.-240 delA, c.*370_371insTG) between dogs and wolves. Interestingly, some of the most striking differences were observed in the coding region: c.141G and c.232T were present as the minor allele in dogs compared to the two wild canid species, while c.259_264 delGCGGCG was only found in wolves and coyotes at allele frequencies of 0.48 and 0.35 respectively.

It has been firmly established that polymorphisms at the regulatory regions flanking the coding sequence of a gene impact the expression and function of the resulting protein. Predictive software allowed us to investigate whether *TRNP1* variants in the three *Canis* species could affect the function of transcription factors and miRNAs that interact with the 5’ end of the gene and 3’ end of the transcript respectively. We focused on those regulatory factors that are expressed in the cerebral cortex, especially those that may be regulating *TRNP1* and other genes involved in cerebellar cortex morphogenesis, and neural precursor cell proliferation.

While the promoter region of a gene can extend thousands of base pairs upstream of the transcription start site, we decided to focus on the 800 nucleotides immediately upstream of the start codon. This proximal promoter region typically contains binding sites for transcription factors that are essential for the proper function for the gene. While a number of transcription binding sites were predicted to be affected by genetic polymorphisms, we focused on those that are known to be expressed in the mammalian embryonic cerebral cortex. Deletion of adenine at -240, which represented the major allele in dogs, but was present at much lower frequencies in wolves and virtually absent in coyotes, was predicted to remove binding sites for REST and ZNF423, both of which regulate neuron differentiation [7]. REST is a transcriptional repressor that is expressed in neural progenitor cells, neurons of the prefrontal cortex, in hippocampal pyramidal neurons, dentate gyrus granule neurons and cerebellar Purkinje and granule neurons [21, 33]. As with TRNP1, the level of expression of REST regulates the migration of radial glia during neocortical development [36]. Most significantly, ChIP-seq experiments indicate that REST binds to the human *TRNP1* promoter in neural progenitor cells [19].

The 3’UTR of transcripts regulate mRNA localization, mRNA stability, and translation into protein. This study showed a high degree of sequence homology between the *TRNP1* 3’UTR across canid species, indicating the importance of conserved sites which likely bind miRNAs and RNA-binding proteins. MicroRNAs play an important role in post-transcriptional gene regulation. For example, miR-128, which is highly expressed in neurons [51], has been shown to regulate the proliferation and neurogenesis of neural precursors, as well as neuronal migration and outgrowth [8, 13, 57]. Specifically, as neurogenesis progresses, neural stem cells reduce miR-128 expression in the developing neocortex [49]. Significantly, the canine homolog cfa-miR-128-1 has been detected in canine cerebrospinal fluid [14]. The sole binding site for miR-128 at the 3’UTR for TRNP1 is predicted to be located at *367-*374 which contains a polymorphic TG repeat (c.*370_*371insTG). Complementary base pairing is only possible if the number of TG repeats is 2 (Additional file 4). All the coyotes and foxes in this study had 3 repeats, as did all the wolves with the exception of an individual originating from the Alaskan interior with a unique (TG)_1_/(TG)_2_ genotype. Interestingly, only 35% of the dogs had the (TG)_2_ allele which would allow miR-128 binding, with, however, 64% of this subgroup being homozygous for this variant.

The length of the TRNP1 protein varies widely across mammalian species, from 219 residues in wolves and coyotes to 233 amino acids in the tiger (*Panthera tigris*) and leopard cat (*Prionailurus bengalensis*) (Additional file 2). This variability is primarily due to a polyalanine repeat (p.Ala84-Ala88) in exon 1, encoded for by GCG repeats located in the c.250-c.264 *TRNP1* sequence in the domestic dog. It is probable that these expansions and deletions arise from replication slippage and/or unequal recombination. This polyalanine repeat is located at the *N*-terminal region of the protein, adjacent to the predicted coiled-coil SNARE domain (located between residues 110 and 171 in the dog). SNARE (soluble *N*-ethylmaleimide-sensitive factor attachment protein receptor) proteins typically consist of a central SNARE motif that is linked to a disordered or low structural complexity *N*-terminal domain which may serve as a binding interface with other proteins [26]. Disordered regions are dynamically flexible and often initiate low-affinity transient interactions with several different proteins. The polyalanine repeat lies within a glycine/alanine-rich domain (that extends from Gly69 to Gly92 in the dog) similar to the one needed for example, for the trans-activation function of the Oct-6 transcription factor which is expressed in glial progenitor and other cells involved in neural development [40]. In general, homopolymeric tracts such as the one observed in TRNP1 are abundant in transcription factors and DNA-binding proteins and play a role in transcriptional repression and protein-protein interactions [2, 3, 28, 40]. The variable polyalanine repeat is predicted to form an alpha-helix in a hydrophobic region at the interface between a disordered region and the coiled-coil domain that comprises the SNARE motif. Comparison of dog (*n* = 5) with red fox (*n* = 8,9,13), as well as in the *Delphinae* (Pacific white-sided dolphin, *n* = 5, vs. common bottle-nosed dolphin, *n* = 9) and *Felidae* (domestic cat, *n* = 6, vs. leopard cat, *n* = 12) demonstrates that increasing polyalanine lengths extend the coiled-coil structure. In addition to differences in polyalanine length and composition between species, within the *Canidae* we observed that this repeat is also polymorphic within wolves, coyotes, and red foxes, but not in dogs. All dogs in the study, irrespective of breed, were observed to have a sequence of 5 alanine repeats. Wolves and coyotes have either a sequence of 3 or 5 repeats, supporting previous observations that even sequences coding for short polyamine domains can be polymorphic [28]. Red foxes may also be subject to significant variability at this locus, as even in the small number of individuals studied, we observed 8, 9 and 13 polyalanine repeats. Conservation of this repeat region across various dog breeds may indicate that expansion or contraction in this region may significantly affect protein function. In genes involved in development, polymorphisms in these polyalanine expansions are associated with various hereditary disorders in humans [27, 41]. Even the addition of a single alanine residue can cause disease [6]. In dogs and other species in the order Carnivora, polyalanine repeats in *FOXI3* have been shown to impact ectodermal development [10], while repeats in *RUNX-2*, *TWIST* and *DIX-2* impact limb and skull morphology [12] and facial length [48]. Thus, changes in the length of these tandem repeats in canine genes regulating growth and development often have the potential to drive rapid interspecific phenotypic evolution [12, 16]. The fact that the *TRNP1* five alanine repeat motif was highly conserved across the dog breeds in this study may underlie a functionally important role for this repetitive sequence.

When aligned with other species representing different families in the order Carnivora, the coding region of TRNP1 in the *Canidae* exhibits a 15-base pair gap at c.174_175, which, in the predicted protein corresponds to the position between Pro58 and Ala59 (Fig. 6), just downstream from the conserved proline-rich region in the *N*-terminal disordered region. In all of the 32 other carnivores for whom transcript or genomic sequences are available, this position is occupied by five amino acids (AWAGS in *Felidae*, *Mustelidae*, *Otariidae*, and *Phocidae* or AWTGS in *Ursidae* or, uniquely, four amino acids (WAGS) in the striped hyena (*Hyaena hyaena*)). Mammalian species belonging to other orders, including primates, also have amino acid sequences that bridge this ‘gap’. A potentially important consequence is that canids, and wolf-like canids in particular have the shortest TRNP1 protein known to exist amongst mammalian species. This potentially significant change in the TRNP1 protein must have occurred early in the evolution of canids, as it is also present in the grey fox, whose lineage is the most primitive amongst the Canidae [31] (Fig. 3, Additional file 2). One has to wonder whether the evolutionary loss of five amino acids been retained throughout the *Canidae* because it has no impact on TRNP1 function, or whether it is crucial to a specific property of the said protein in this family of canid species.

## Conclusions

The TRNP1 gene, expressed in several regions of the brain in domestic dogs, displays a number of potentially functionally significant genetic polymorphisms in the coding and flanking regulatory regions. While some of these variants are shared with other canid species, others are species-specific. A polyalanine repeat, which is predicted to affect the formation of alpha helices and coiled-coils, was found to be polymorphic in wolves, coyotes and red foxes, but not in the dog breeds investigated. Compared to other carnivores and mammalian species in general, the *Canidae* species all have a shorter TRNP1 protein due to a five-amino acid ‘gap’ in the *N*-terminal domain.

### Supplementary Information

**Additional file 1. **Details of individual pure-bred dogs, wolves, coyotes and foxes used in the study.

**Additional file 2. **Accession numbers for Canis lupus familiaris and Vulpes vulpes polymorphisms found in the study.

**Additional file 3.**

**Additional file 4. **Mirtarget prediction of binding of cfa-miR-128 To dog TRNP1 3'UTR with c.*370_371insAG ALLELE.

## Data Availability

All polymorphism locus and population data for canids with GenBank reference genomes, that is, dog (GCA_011100685.1) and red fox (GCA_003160815.1), were deposited in the European Variation archive https://www.ebi.ac.uk/eva/, project numbers PRJEB55868 (dog), PRJEB55814 (red fox). The TRNP1 partial transcript sequences were submitted to GenBank https://www.ncbi.nlm.nih.gov/genbank/ and designated by accession numbers OQ191934 and OQ191935.
